# Supplementary figures and images for: Spatial distribution of advanced stage diagnosis and mortality of breast cancer: Socioeconomic and health service offer inequalities in Brazil
Source: PLoS One. 2021 Feb 3;16(2):e0246333. doi: 10.1371/journal.pone.0246333 (PMC7857585; doi:10.1371/journal.pone.0246333)

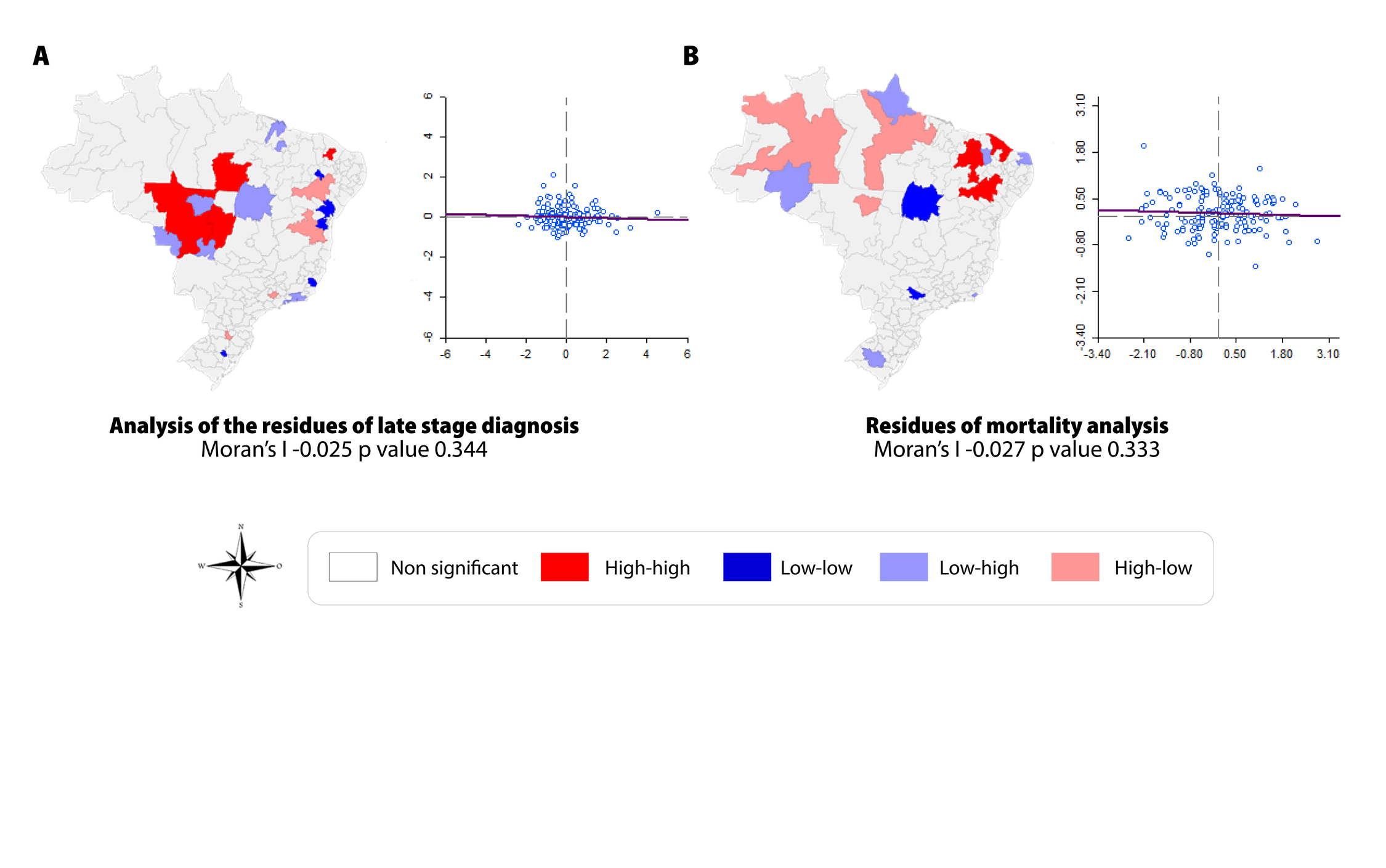

Supplement: S1 Fig — (TIF) [file pone.0246333.s001.tif]
