# Supplementary material for: Spatial distribution of advanced stage diagnosis and mortality of breast cancer: Socioeconomic and health service offer inequalities in Brazil
Source: PLoS One. 2021 Feb 3;16(2):e0246333. doi: 10.1371/journal.pone.0246333 (PMC7857585; doi:10.1371/journal.pone.0246333)
Supplement: S1 Table — (DOCX) [file pone.0246333.s002.docx]

**S1 Table.** Comparative data between the spatial regressions of the proportion of late stage diagnosis and adjusted mortality rates for breast cancer.

|  | **Constant** | **Lambda** | **Akaike’s criterion** | **R Squared** |
| --- | --- | --- | --- | --- |
| **Late Stage Diagnosis of Breast Cancer** | | | | |
| **Spatial Error Model** | 16.24 | 0.65 | 1074.59 | 0.525 |
| **Spatial Lag Model** | - 5.08 | 0.61 | 1074.74 | 0.521 |
| **Classic regression** | - 1.85 | --- | 1121.51 | 0.295 |
| **Adjusted mortality rates for breast cancer** | | | | |
| **Spatial Error Model** | - 14.43 | 0.67 | 675.07 | 0.657 |
| **Spatial Lag Model** | - 3.57 | 0.35 | 699.22 | 0.573 |
| **Classic regression** | - 3.06 | --- | 712.87 | 0.516 |
